# Knowledge, attitude and practice on antimicrobial use and antimicrobial resistance among competent persons in the community pharmacies in Bhutan

**DOI:** 10.3389/fpubh.2023.1113239

**Published:** 2023-06-15

**Authors:** Jigme Tenzin, Kinley Penjor Tshomo, Sonam Wangda, Wangdi Gyeltshen, Gyem Tshering

**Affiliations:** ^1^Drug Regulatory Authority, Thimphu, Bhutan; ^2^Ministry of Health, Thimphu, Bhutan

**Keywords:** competent person, community pharmacy, antimicrobial use, antimicrobial resistance, knowledge, attitude, practice, Bhutan

## Abstract

**Introduction:**

Since the discovery, antimicrobials have been used to treat variety of infections both in humans and animals caused by microbes. However, with the increasing use, microbes developed resistance to the antimicrobials and many of the antimicrobials became ineffective against certain microbes. Many factors are reported to contribute to the resistance of microbes to antimicrobials. One contributing factor is the misuse and overuse of antibiotics which mainly occur due to the lack of knowledge, careless attitudes, and incorrect practices about use of antibiotics.

**Methods:**

This cross-sectional survey study was conducted among the competent persons (CP) in the community pharmacies in Bhutan, with the aim to assess their knowledge, attitude and practice (KAP) on antimicrobial use (AMU) and antimicrobial resistance (AMR).

**Results:**

Results from the survey revealed that the competent persons had good level of knowledge about antimicrobial use and antimicrobial resistance. They also had favourable attitude towards antimicrobial resistance and rational use of antimicrobials. Their knowledge and attitude had led to good practices while dispensing antimicrobials from their pharmacies. However, almost all of them had never had any opportunity to take part in activities related to antimicrobial use and antimicrobial resistance that were organized by the public sector. Many of them did not even hear or know about the existence of the policies on use of antimicrobials or on curbing antimicrobial resistance in the country.

**Conclusion:**

Involvement of the community pharmacies through trainings and participations in policy making processes is seen as a vital mechanism that can eventually help achieve the goals in the national drive towards reducing antimicrobial resistance.

## 1. Background

Antimicrobials have been used to treat a variety of infections both in humans and animals caused by microorganisms. Since the first discovery of penicillin by Sir Alexander Fleming in 1928 (1), many other antimicrobials were introduced thereby leading to the quick treatment of various kinds of infectious diseases (2, 3). The development and introduction of new antimicrobials had gradually increased until the 1960s, after which, hardly any new antimicrobials have been introduced in the market (4, 5). On the other hand, the persistent misuse of antimicrobials in human and animal health has led to the emergence of antimicrobial resistance (AMR) which posed a threat to global public health (6). In fact, Sir Fleming was one among the first persons to warn about possible resistance to penicillin if used in little doses or for inadequate periods (7). The inappropriate and irrational antimicrobial consumption are potential causes of the increasing prevalence of AMR (8). Tackling the emerging AMR has become an urgent priority worldwide (9). It has been reported that around 700,000 people die from AMR worldwide every year and this figure is estimated to reach 10 million by 2050, if effective countermeasures are not put in place (10).

A systematic review and meta-analysis has shown the inappropriate use of antibiotics by the general population, as seen in behaviors such as purchasing antibiotics without a prescription from pharmacies and not completing the entire course of antibiotics as prescribed by the physicians which causes the microbes to become resistant leading to AMR (11). Another major challenge particularly in developing countries is self-medication with antibiotics. A systematic review reported that in the Southeast Asian region, the prevalence of self-medication of antibiotics is around 50% (10). Misuse and overuse of antibiotics could occur due to a lack of knowledge, careless attitudes, and incorrect practices about antibiotics (11).

Bhutan is also facing challenges of AMR just like any other developing country. There are reported evidences of resistance of many microbes. A study by Tshokey et al. reported resistance of *Neisseria gonorrhoea* against ciprofloxacin (85.1%), penicillin (99.2%), tetracycline (84.8%) and nalidixic acid 99.7% (12). Another report from urine and blood samples received for microbiological investigation showed that *E. coli* was resistant to ampicillin (73.5%) and 3rd gen cephalosporins (73.2%), *K. pneumoniae* was resistant to 3rd gen cephalosporins (78.2%) and aminoglycosides (63.9%), and *S. aureus* was resistant to penicillin (98.3%) (13). What is more concerning is that resistance to high level antibiotics have also been detected here. What factors are contributing to the trend of resistance in this country remains to be sorted out. Nevertheless, since antimicrobials are readily available from the community pharmacies across the country despite the strict legislations on requirements of prescriptions for dispensing antimicrobials, it is fairly arguable that the easy access might, in part, be adding to the increasing trend of AMR in the country.

Taking into account the possible contributing factors toward misuse of antimicrobials and AMR, this cross-sectional study is aimed at assessing the knowledge, attitude and practice of the competent persons (CPs) in the community pharmacies on antimicrobial use (AMU) and AMR. As per Bhutan Medicines Rules and Regulation 2019 “CP refers to any person who possesses the requisite qualifications and practical experience prescribed by the Bhutan Medicines Board and is approved to undertake retail sale and dispensing of Medicinal Products.”

## 2. Materials and methods

### 2.1. Study design and setting

**Figure 1:**
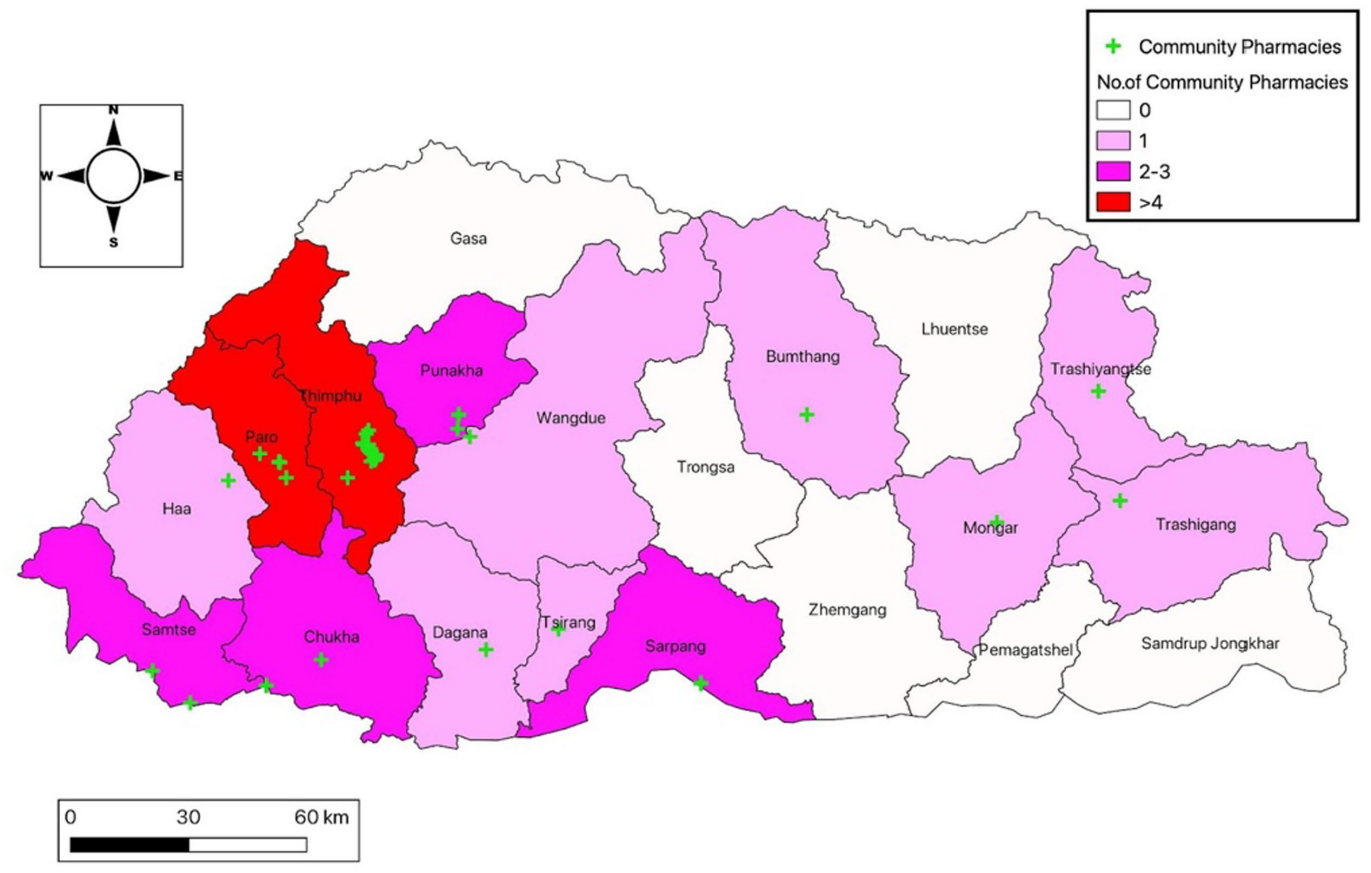
Map of Bhutan showing the number and distribution of community pharmacies involved in the study.

A descriptive cross-sectional survey was carried out from October to November 2021, in 55 operational community pharmacies across the country, Figure 1. The survey questionnaire was developed by a team of experienced pharmacists through literature review of previous studies on similar topics in comparable settings. The finalized questionnaire had three main sections viz.: knowledge, attitude, and practice (KAP) of the competent persons with regard to AMU and AMR. Seventeen regulatory officials (pharmacists and pharmacy technician) from the Drug Regulatory Authority (DRA) and 2 district hospital pharmacists were trained on the use of the survey questionnaires and deputed for the survey. A total of 58 CPs engaged in sales of medicinal products from the 55 community pharmacies were interviewed.

### 2.2. Data analysis

Data was collected using Epicollect 5 (Imperial College of London, England). Data validation and analysis were performed using SPSS software (version 21.0, IBM Corp., New York, United States) and MS excel. Results are presented in frequencies and percentages. The geospatial information of community pharmacies was established using QGIS LTR 3.16. Descriptive statistics were performed by calculating the proportions of frequencies to describe the demographic characteristics and KAP score. For the knowledge and the practice section, a score of “1” was allotted for the correct answer and “0” for the wrong answer, based on the regulatory requirements and international practices. The total score was then added and those who scored above the mean was categorized as “good” and those who scored equal to and below the mean were defined as “poor” in terms of knowledge and practice on AMU and AMR.

The attitude section had parameters whereby the responses can be easily differentiated into having a “favorable” or “unfavorable” attitude toward AMU and AMR.

### 2.3. Ethical approval

Since the survey was carried out as part of a routine regulatory activity of the DRA of Bhutan, ethics approval was not required by the local ethics board. Administrative permission was granted by the DRA to use the data for publication. No identifiable variables were collected and all participants provided informed consent after reading and agreeing to the information and consent form on the first page of the online survey.

## 3. Results

### 3.1. Demographics characteristics

Among the 58 CPs interviewed, more than half of the respondents (62.07%, *n* = 36) were male and 37.93% (*n* = 22) were female. Most of the survey participants fell under the age range of 26–35 (41.3%, *n* = 24). Almost half of the CPs (46.55%, *n* = 27) had less than 5 years of experience and the majority of them had certificate level (37.93%, *n* = 22) and bachelor’s degree qualifications (32.76%, *n* = 19) (Table 1).

**Table 1:** Demographic characteristics of the CPs.

Sl. No.	Variables		CPs	
			*n*	%
1	Gender	Male	36	62.07
		Female	22	37.93
2	Age (years)	≤25	7	12.07
		26–35	24	41.38
		36–45	5	8.62
		46–55	8	13.79
		56–65	9	15.52
		≥66	5	8.62
3	Years of experience	0–5	27	46.55
		6–10	12	20.69
		11–15	7	12.07
		16–20	3	5.17
		21–25	2	3.45
		26–30	3	5.17
		≥31	4	6.90
4	Level of education	Master’s degree	1	1.72
		Bachelor’s Degree	19	32.76
		Diploma	11	18.97
		Certificate	22	37.93
		Others	5	8.62

### 3.2. Competent persons’ knowledge on AMU and AMR

**Figure 2:**
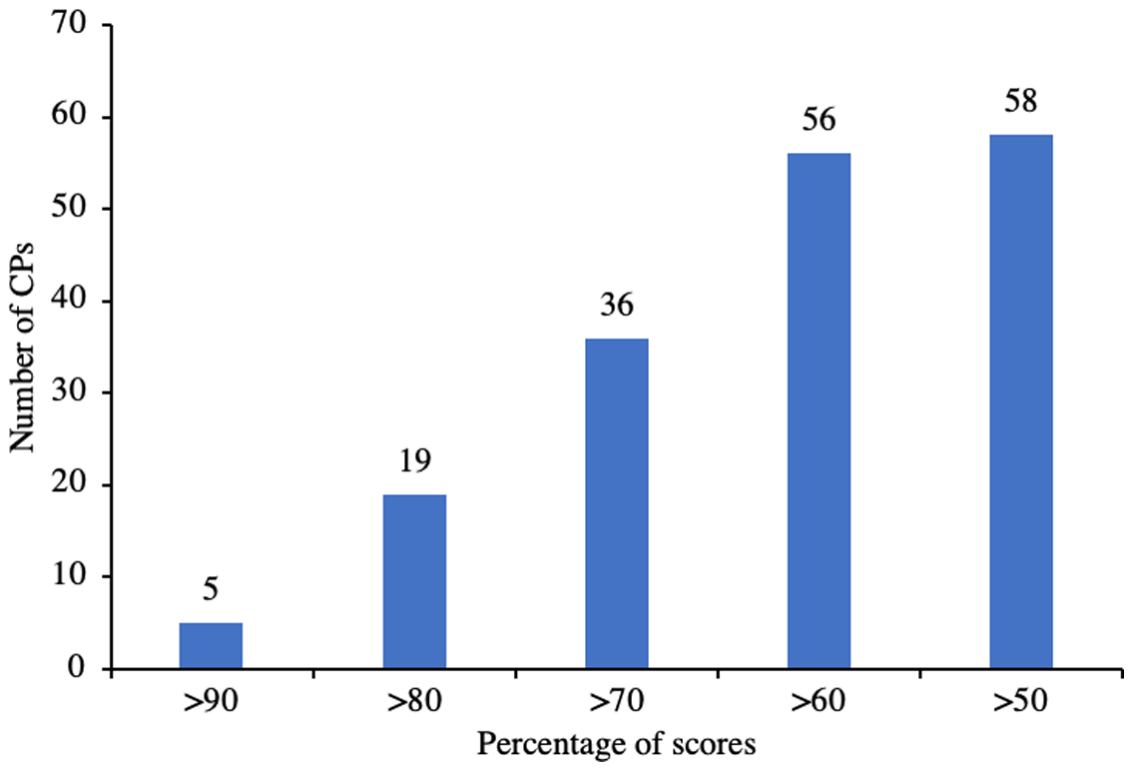
Number of competent persons with score ranges in the overall knowledge section.

The overall knowledge scores of the CPs were found to be above 50%. Out of that, 89.66% (*n* = 56) CPs had scored >60, 62.07% (*n* = 36) hxad scored >70, 32.76% (*n* = 19) had scored >80 and 8.62% (*n* = 5) had scored >90%, Figure 2. When given the choice to select the antimicrobials from a list, majority of the CPs (29.31%, *n* = 17) chose the wrong option, while many (27.59%, *n* = 16) selected only 1 option. Only a small number of CPs (6.9%, *n* = 4) selected all the 5 antimicrobials correctly from the list, Supplementary Figure S1. Majority of the CPs (70.69%, *n* = 41) were aware that antibiotics are not effective against the common cold or flu. Most of them (96.55%, *n* = 56) were also aware that inappropriate use of antimicrobials would lead to AMR. Almost all the CPs (91.38%, *n* = 53) knew that antibiotics should not be stopped soon after the disease symptoms were resolved. All of them were aware that they cannot dispense antibiotics for similar previous infections without prescription and also that SOPs were required for storage and dispensing of medicines. Most of them (87.93%, *n* = 51) knew that they had to retain a copy of prescription for every antibiotic sold. However, quite a huge number of them (43.10%, *n* = 25) did not know that they were not allowed to sell tropical antibiotics without a prescription, while a few (5.17%, *n* = 3) were not at all aware of such legislation, Table 2.

**Table 2:** Competent Persons’ knowledge on AMU and AMR.

Questions	Frequency	Percentage
1. Are you aware of Antimicrobial Resistance (AMR)?		
Yes	54	93.10
No	4	6.90
Not sure	0	0
2. Are antibiotics effective against the common cold or flu?		
Yes	15	25.86
No	41	70.69
Not sure	2	3.45
3. Does inappropriate use of antimicrobials lead to AMR?		
Yes	56	96.55
No	0	0
Not sure	2	3.45
4. Are you allowed to sell topical antibiotics without prescriptions?		
Yes	25	43.10
No	30	51.72
Not sure	3	5.17
5. Do the use of topical antimicrobials contribute to AMR?		
Yes	48	82.76
No	4	6.90
Not sure	6	10.34
6. Should antibiotics be stopped soon after symptoms are resolved?		
Yes	5	8.62
No	53	91.38
Not sure	0	0
7. Can you dispense antibiotics for similar previous infections without prescription?		
Yes	58	100
No	0	0
Not sure	0	0
8. Are you aware of the requirement of SOP for storage and dispensing of medicines?		
Yes	58	100
No	0	0
Not sure	0	0
9. Are you aware of the requirement to retain a copy of prescription for every antibiotic sold?		
Yes	51	87.93
No	7	12.07
Not sure	0	0
10. Are you aware of the national antibiotic guideline?		
Yes	38	65.52
No	15	25.86
Not sure	5	8.62

Major sources of information for the CPs included Health Professionals (36), Internet (36), and Bhutan National Formulary (35) based on the frequency of sources selected, Supplementary Figure S2.

In terms of possible interventions by the CPs for patients with minor side effects, referral to hospital was the most frequently chosen option (36), followed by reassurance (15) and investigation of the severity, and advising accordingly (11), Supplementary Figure S3. The most frequently chosen intervention was the same even for patients with serious side effects, Supplementary Figure S4.

### 3.3. Competent persons’ attitudes toward AMU and AMR

More than half (63.97%, *n* = 40) of the 58 CPs interviewed accepted that AMR is a global issue, and 62.07% (*n* = 36) recognized AMR as an issue of concern in Bhutan. Half of the CPs (*n* = 29) felt that the patients coming to their pharmacy had very little understanding on both antimicrobials and AMR, while 18.97% (*n* = 11) felt that the patients do not have any knowledge on antimicrobials and 34.48% (*n* = 20) of them are of the opinion that the patients visiting their pharmacies have no understanding on AMR at all. Majority of the CPs (86.21%, *n* = 50) felt that it is crucial to advise the patients on complying with treatment when antimicrobials are dispensed and likewise, 77.59% (*n* = 45) of the CPs felt that patients value their counseling on rational use of antimicrobials.

Close to half of the CPs (41.38%, *n* = 24) stated that they did not have opportunities to attend Continued Medical education (CME) on AMR and all of them were willing to attend CMEs, conference and workshops on antimicrobials and AMR for better understanding and practice, as majority of them (94.83%, *n* = 55) felt that CPs played an important role in tackling AMR, Table 3. Dispensing antimicrobials only on prescription was the most frequently chosen role of CPs in tackling AMR, followed by educating patients on rational use of antimicrobials, Supplementary Figure S5.

**Table 3:** Competent Persons’ attitude towards AMU and AMR.

Characteristics	Frequency	Percentage
1. AMR is a global public health problem		
Strongly agree	40	68.97
Agree	17	29.31
Not sure	1	1.72
Disagree	0	0
Strongly disagree	0	0
2. AMR is an issue of concern in our country		
Strongly agree	36	62.07
Agree	19	32.76
Not sure	3	5.17
Disagree	0	0
Strongly disagree	0	0
3. It is important to advise patients about complying with the treatment when antimicrobials are dispensed		
Strongly agree	50	86.21
Agree	7	12.06
Not sure	1	1.72
Disagree	0	0
Strongly disagree	0	0
4. In your opinion, do people coming to your pharmacy know about antimicrobials?		
To a great extent	0	0
Somewhat	18	31.03
Very Little	29	50
Not at all	11	18.97
5. Do you think people buying antimicrobials have a good understanding of AMR?		
To a great extent	1	1.72
Somewhat	8	13.79
Very Little	29	50
Not at all	20	34.48
6. Do patients come to get antibiotics without a valid prescription?		
Often	16	27.59
Never	3	5.17
Sometimes	39	67.24
7. Do you feel that there are adequate policies and interventions in place to curb issues of AMR in the country?		
Yes	29	44.83
No	9	15.52
Not sure	23	39.66
8. Did you ever feel compelled by stakeholders (for example doctors, owners of the pharmacy, patients) to sell antimicrobials without prescriptions?		
Yes	28	48.28
No	30	51.72
Not sure	0	0
9. Do you think you have opportunities to attend CMEs on AMR?		
Yes	34	58.62
No	24	41.38
Not sure	0	0
10. Are you willing to attend CME, conferences and/or workshops on antimicrobials and AMR for better understanding and practice?		
Yes	58	100
No	0	0
Not sure	0	0
11. Do you think the Competent Persons play an important role in tackling AMR		
Yes	55	94.83
No	1	1.72
Not sure	2	3.45
12. Do you think patients value your counseling on rational use of antimicrobials?		
Yes	45	77.59
No	3	5.17
Not sure	10	17.24

CPs felt that incomplete courses of antimicrobials contributed most to AMR, followed by self-medication and use of antimicrobials when not indicated, Supplementary Figure S6. They were interested in learning about new antibiotics followed by AMR and national guidelines, Supplementary Figure S7.

### 3.4. Competent persons’ AMU and AMR practices

**Figure 3:**
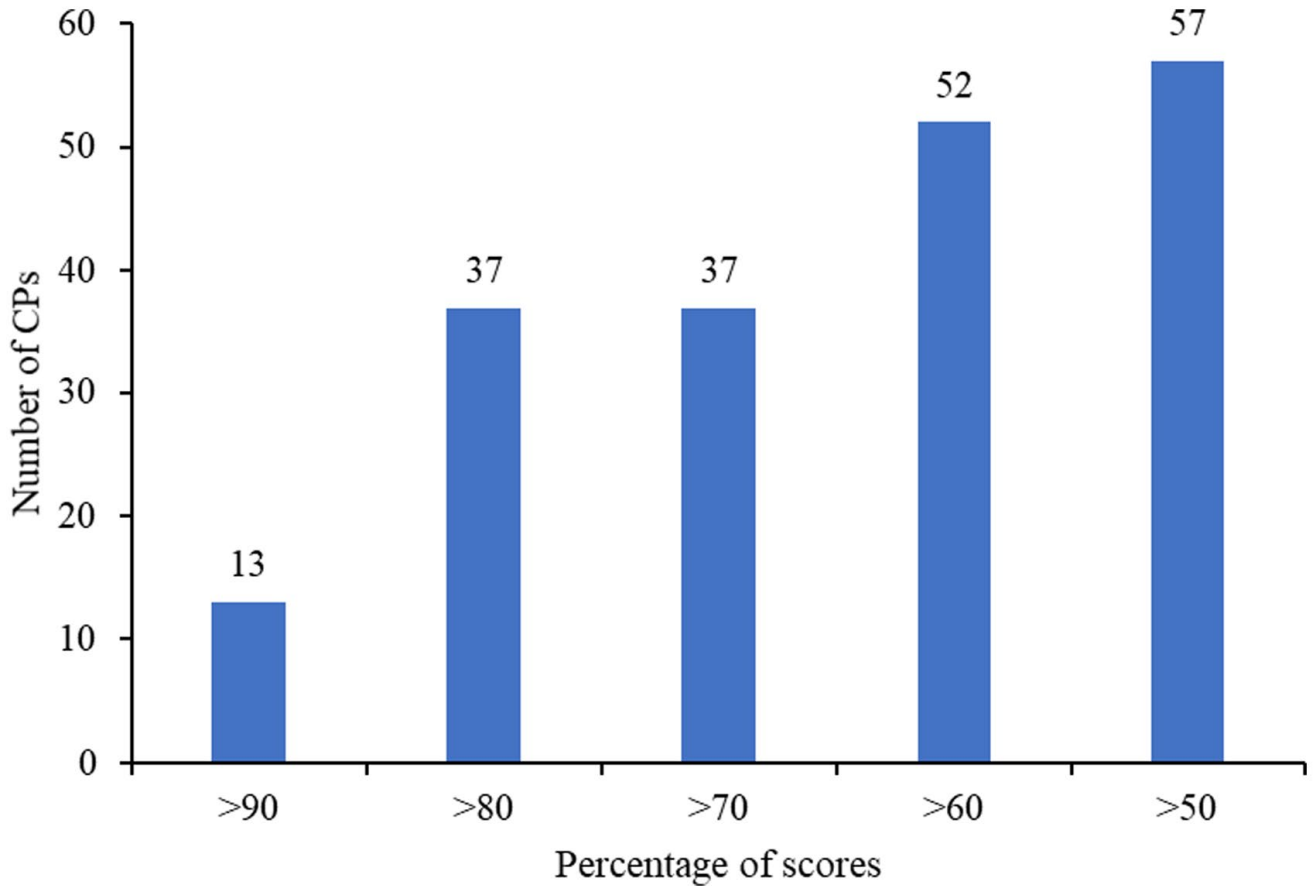
Number of competent persons with score ranges in the overall practice section.

**Figure 4:**
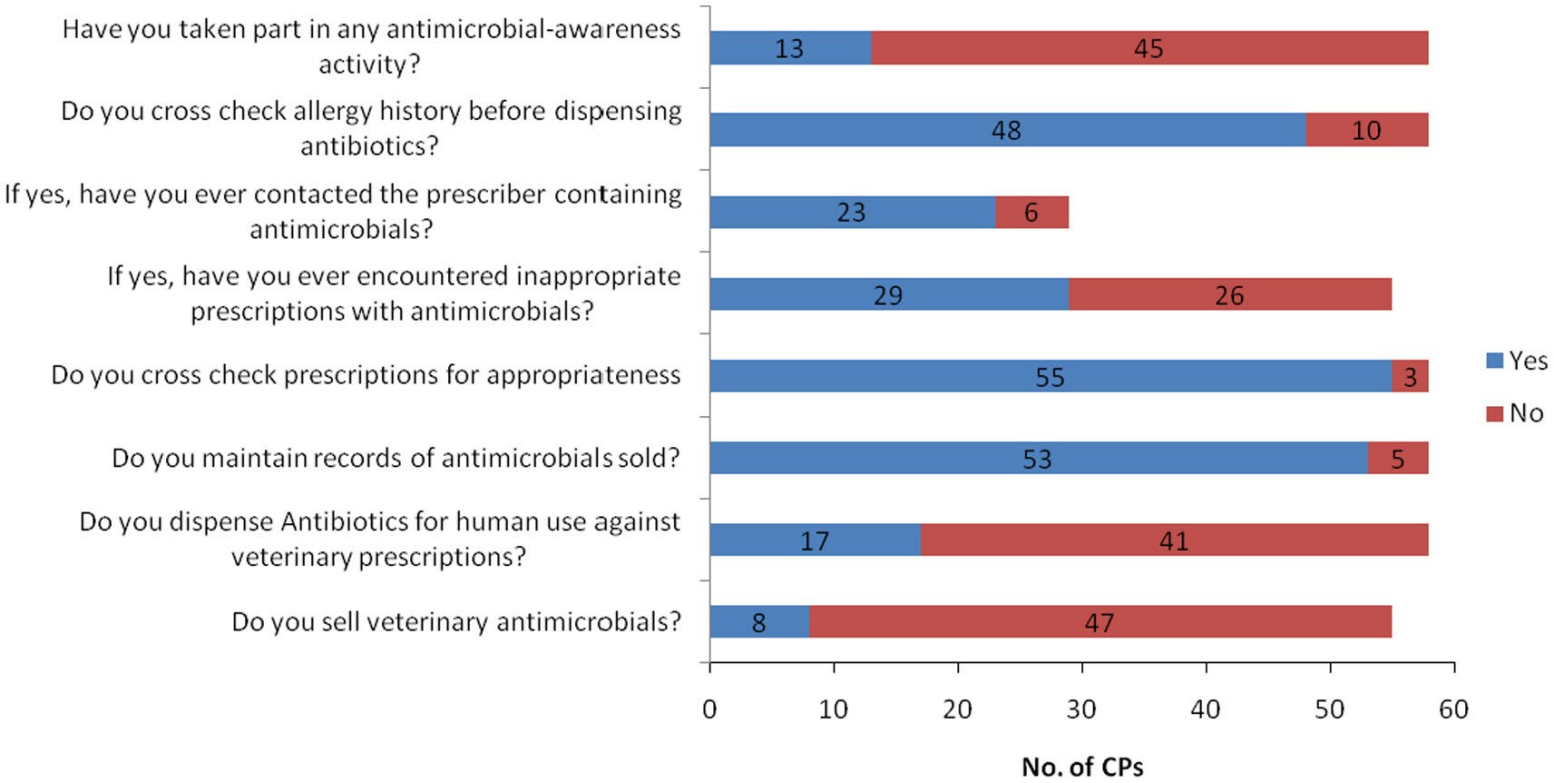
Competent persons’ AMU and AMR practices.

Almost all of the CPs (98.28%, *n* = 57) had scored above the mean in the overall practice scores, while 89.66% (*n* = 52), 63.79% (*n* = 37), 63.79% (*n* = 37) and 22.41% (*n* = 13) of them had scored more than 60, 70, 80 and 90%, respectively, Figure 3. More than 90% (*n* = 53) of CPs out of 58 maintained records whereas 8.62% (*n* = 5) did not maintain records of antimicrobials sold. Majority of the CPs (94.83%, *n* = 55) checked the appropriateness of prescription, specifically rationality of prescription, while 5.17% (*n* = 3) did not. From 58 CPs who cross-checked the prescriptions, 52.73% (*n* = 29) of them reported receiving inappropriate prescriptions with antimicrobials, and out of that, 79.31% (*n* = 23) of them communicated to the prescriber regarding the inappropriate prescriptions. While 22.41% (*n* = 13) of CPs had participated in antimicrobial-awareness activities, majority of them 77.59% (*n* = 45) did not get any opportunities to take part in such activities. The community pharmacies in the country authorized by the DRA are also allowed to sell veterinary medicines in addition to human allopathic medicines. Out of 55 community pharmacies involved in the survey, 14.55% (*n* = 8) of community pharmacies dealt with both human and veterinary antimicrobials while the remaining pharmacies dealt only with human antimicrobials, Figure 4.

## 4. Discussion

This study was conducted to assess the knowledge, attitude and practice of AMU and AMR among competent persons working in community pharmacies across Bhutan. The secondary aim is to establish a baseline data source and provide evidence for appropriate interventions. A total of 58 CPs from 55 community pharmacies were interviewed using survey questionnaires.

Our results showed that the CPs at the community pharmacies had a good overall knowledge on AMU and AMR with 100% of them securing more than the mean score. Almost all of them (93.10%, *n* = 54) were aware of AMR. However, the knowledgeable rating on the overall scores may not imply that all the CPs were well aware of the AMU and AMR. When we analysed the responses to the survey questions individually, we found out that quite a huge number of CPs have given the wrong responses to some of the critical questions. With regard to the law of selling antibiotics without prescriptions, many of them (43.10%, *n* = 25) were not aware that they were not allowed to sell antibiotics without prescriptions, while a few (5.17%, *n* = 3) were not at all aware of that law. This is comparable to a study conducted in Northeast China where nearly half of the participants (40.6%, *n* = 162) sell antimicrobials without a prescription to patients occasionally (6). Nevertheless, the majority of the CPs (70.69%, *n* = 41) were aware that antibiotics are not indicated for common cold or flu, indicating a higher level of knowledge compared to a study conducted in Indonesia where more than half of respondents (73.12%, *n* = 420) assumed that antibiotics can be used to treat virus infection (11). Almost all of them (91.38%, *n* = 53) also knew that antibiotics should not be stopped soon after disease symptoms were resolved. In line with earlier studies (14, 15), most of the CPs (96.55%, *n* = 56) agreed that inappropriate use of antimicrobials would lead to AMR.

Reports suggest that AMR is mainly driven by misuse of antibiotics due to lack of knowledge on antimicrobials (16, 17). While AMR has been an issue globally, the majority of CPs (62.07%, *n* = 36) strongly agreed that it is an issue in our country as well. In this context, 50% of the CPs (*n* = 29) agreed that one of the leading factors could be the lack of knowledge or little understanding on AMU and AMR by the general public as evidenced by the findings from this study. In addition, the majority of CPs also agreed that the community pharmacies played a vital role in tackling AMR in the community. In agreement to the responses from the CPs (94.83%, *n* = 55), ensuring antimicrobials are dispensed only on prescription, educating the patients on rational use of antimicrobials, and also educating the general public on AMR could be core components while dispensing. And this looks possibly successful since most of the patients (77.59%, *n* = 45) coming to the pharmacies were found to value the counseling and instructions given by the CPs.

Considering the overall good knowledge and attitude toward AMU and AMR of the CPs in the community pharmacies as revealed by this study, the practice toward rational AMU and AMR awareness were found comparatively adequate with compliance above the mean value. However, as revealed by this study, there are a number of CPs who do not really understand the concept of AMU and AMR, although the majority had satisfyingly adequate overall knowledge and practice on AMU and AMR. Hence, it is impossible to rule out that the sale of antimicrobials by these CPs might be directly or indirectly contributing to the issue of AMR in the country. Providing specific trainings and orientations on the related topics would help build their knowledge and confidence in antimicrobial fields. Moreover, the majority (77.59%, *n* = 45) of them did not take part in any antimicrobial-awareness activities given the limited opportunity and platform. Hence it is evident that to improve the public-private partnership toward curving the issues of AMR, community pharmacies should be given priority while making policies with regard to AMU and AMR.

### 4.1. Limitations

Besides our best of efforts, this study does have some limitations. The survey was carried out by the officials of the DRA, which could have caused the respondents to give favorable answers in topics related to regulations. However, the respondents were thoroughly oriented on the objective of the survey and assured of no implications to whatever response they provide during the survey. Other potential limitations include the small sample size and the unequal distribution of the community pharmacies throughout the country.

## 5. Conclusion

This study is the first of its kind in Bhutan, assessing the knowledge, attitude and practices of the CPs in the community pharmacies on antimicrobial use and antimicrobial resistance. Results showed that the CPs had good level of knowledge on AMU and AMR. They had favorable attitude toward AMR and rational use of antimicrobials. Even in practice, they are shown to be following the good practices while dispensing antimicrobials to the patients. However, most of them did not get any opportunities to participate in the awareness activities organized by the public sector. Many of them were not aware of the existence of the policies on reducing AMR. Since antimicrobials are readily available from the community pharmacies, the CPs may be recognized as key players in the national drive toward curbing AMR in the country.

## Data availability statement

The original contributions presented in the study are included in the article/Supplementary material, further inquiries can be directed to the corresponding authors.

## Ethics statement

Ethical review and approval was not required for the study of human participants in accordance with the local legislation and institutional requirements. Written informed consent from the patients/participants was not required to participate in this study in accordance with the national legislation and the institutional requirements.

## Author contributions

WG, SW, JT, and GT planned the overall survey methodology with WG as the approving official. JT, KPT, and GT carried out the survey along with other officials. JT, KPT, and GT drafted the manuscript. SW and WG reviewed the manuscript. SW sought funding for the survey. All authors contributed to the article and approved the submitted version.

## Funding

This study was funded by the Fleming Fund Project, under the Ministry of Health, Bhutan.

## Conflict of interest

The authors declare that the research was conducted in the absence of any commercial or financial relationships that could be construed as a potential conflict of interest.

## Publisher’s note

All claims expressed in this article are solely those of the authors and do not necessarily represent those of their affiliated organizations, or those of the publisher, the editors and the reviewers. Any product that may be evaluated in this article, or claim that may be made by its manufacturer, is not guaranteed or endorsed by the publisher.
