# Supplementary material for: Knowledge, attitude and practice on antimicrobial use and antimicrobial resistance among competent persons in the community pharmacies in Bhutan
Source: Front Public Health. 2023 Jun 15;11:1113239. doi: 10.3389/fpubh.2023.1113239 (PMC10308045; doi:10.3389/fpubh.2023.1113239)
Supplement: Supplementary file 1 [file Table_1.DOCX]

Supplementary data for Knowledge, Attitude and Practice on Antimicrobial Use and Antimicrobial Resistance among Competent Persons in the Community Pharmacies in Bhutan

**Results**

***Competent Persons’ knowledge on AMU and AMR***

**
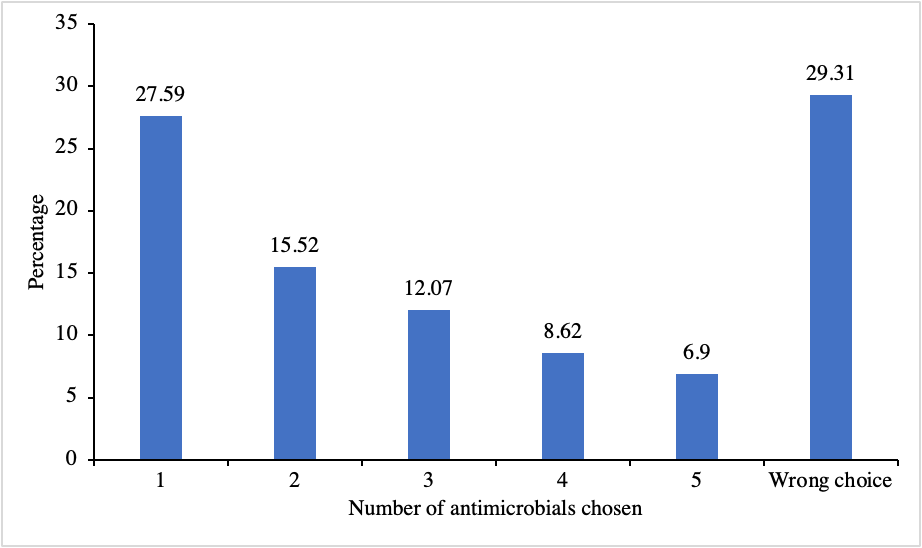
**

Figure s1: Percentage of CPs on choosing the antimicrobials from the list provided

**
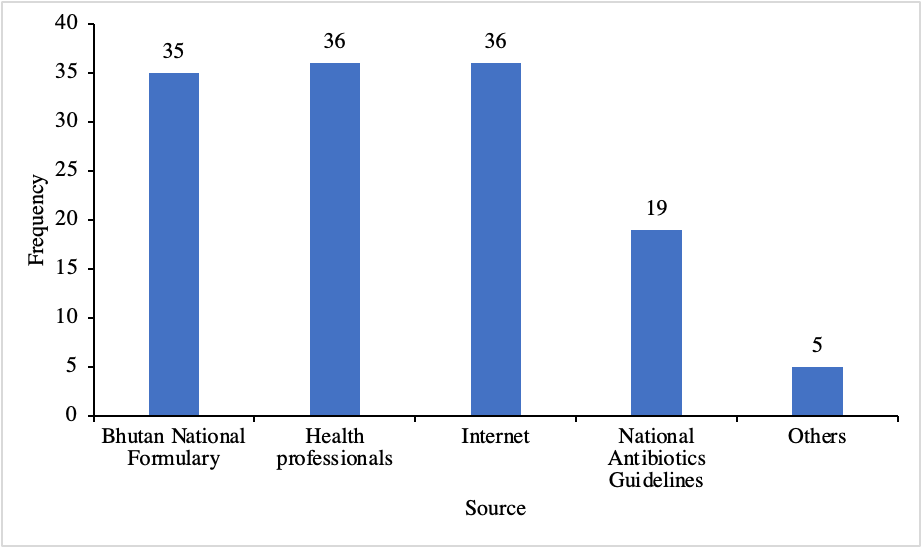
**

Figure s2: Sources of information for the CPs

**
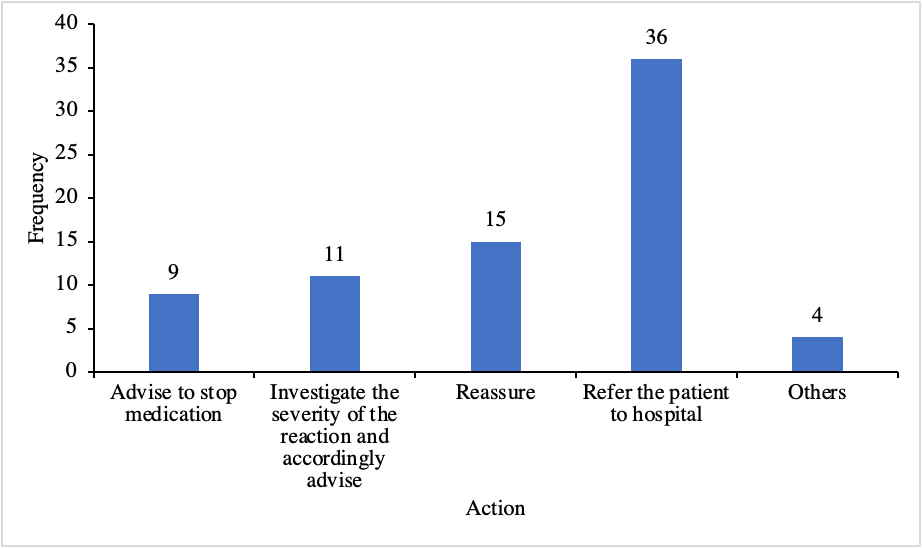
**

Figure s3: Possible interventions against patients with minor SEs

**
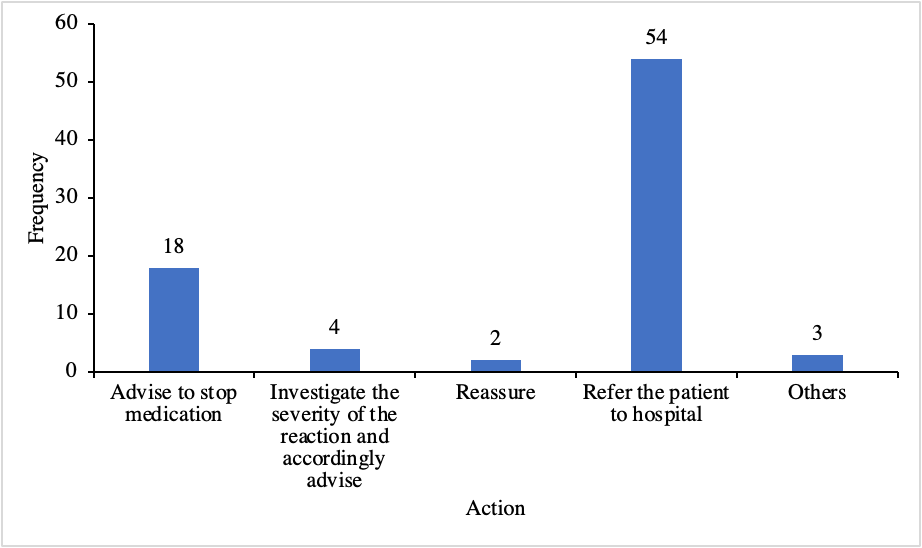
**

Figure s4: Possible interventions against patients with serious SEs

***Competent Persons’ attitudes toward AMU and AMR***

**
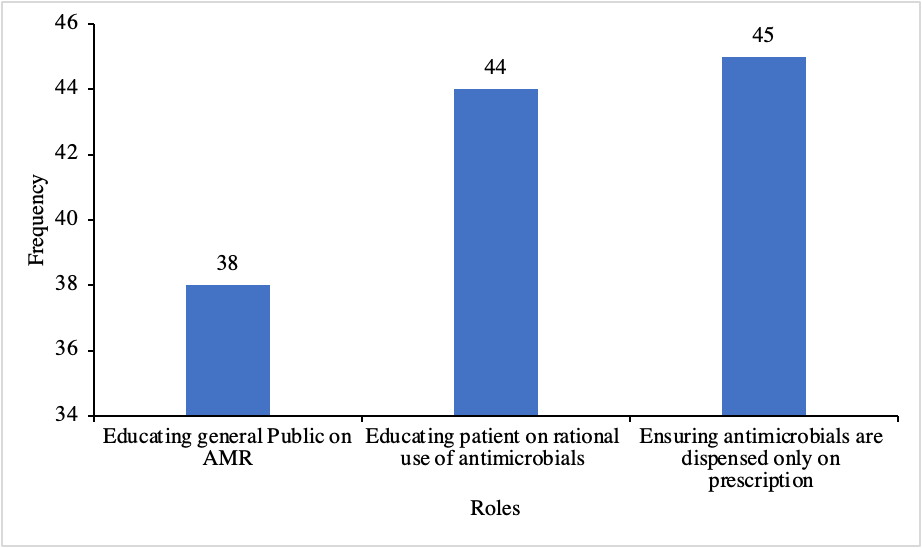
**

Figure s5: Possible roles of CPs in tackling AMR

**
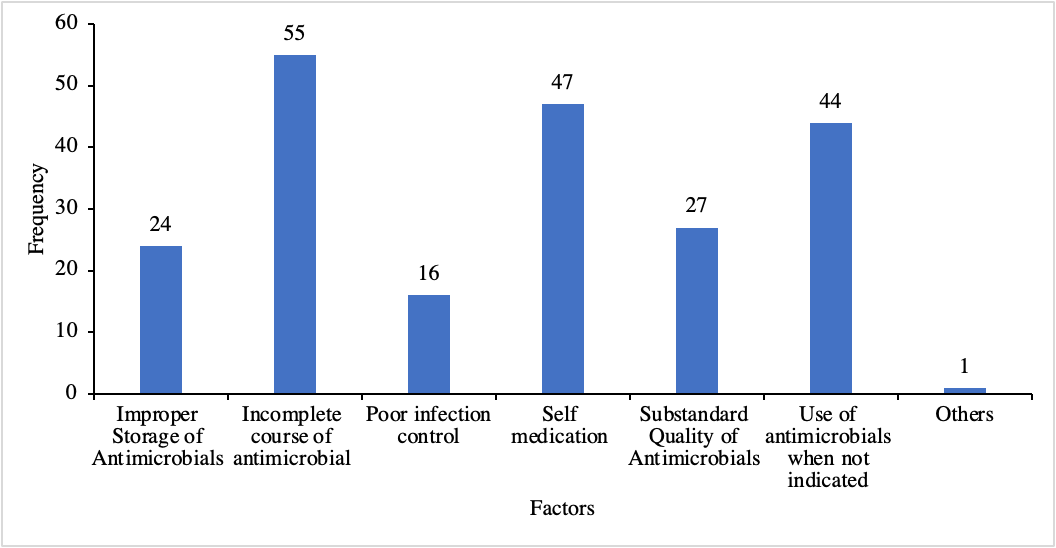
**

Figure s6: Predicted factors contributing to AMR

**
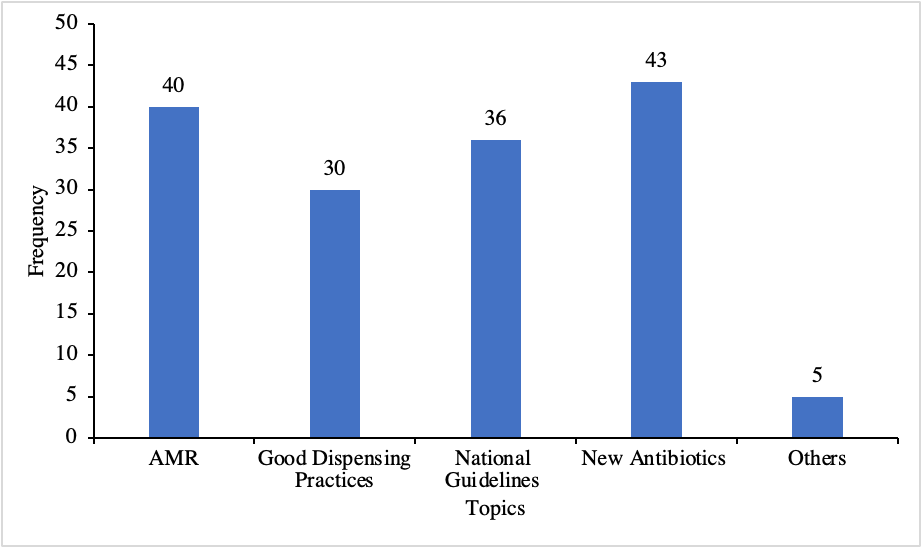
**

Figure s7: Topics of interest for the CMEs that the CPs would like to avail
